# Dog ecology and rabies control including canine vaccination coverage: Impacts from a survey in Madagascar

**DOI:** 10.1371/journal.pone.0302690

**Published:** 2024-05-09

**Authors:** Blaise Rajoromanana, Gabriel Nyirenda, Glenn T. Edosoa, Radonirina L. Andrianasolo, Sylvie Rietmann, Florian Marks, Raphaël Rakotozandrindrainy, Andrea Haselbeck, Paule-Aimée Ralison Farasolo

**Affiliations:** 1 Faculté de Médecine Vétérinaire, Université d’Antananarivo, Antananarivo, Madagascar; 2 International Vaccine Institute, Seoul, South Korea; 3 Ministère de la Santé Publique, Antananarivo, Madagascar; 4 Université d’Antananarivo, Antananarivo, Madagascar; 5 Cambridge Institute of Therapeutic Immunology and Infectious Disease, University of Cambridge School of Clinical Medicine, Cambridge Biomedical Campus, Cambridge, United Kingdom; 6 Faculté de Science, de Technologie et de l’environnement, Université de Mahajanga, Mahajanga, Madagascar; University of Oklahama Norman Campus: The University of Oklahoma, UNITED STATES

## Abstract

**Background:**

Rabies virus (RABV*;* species *Lyssavirus rabies)* is causing one of the oldest zoonotic diseases known to mankind, leading to fatal encephalomyelitis in animals and humans. Despite the existence of safe and effective vaccines to prevent the disease, an estimated 99% of human rabies deaths worldwide are caused by dog-mediated rabies with children at the highest risk of infection. Rabies has been endemic in Madagascar for over a century, yet there has been little research evaluating local knowledge and practices impacting on the rabies control and prevention. Thus, this study was undertaken to better understand the dog ecology including canine vaccine coverage and to assess knowledge and practices of dog owners and veterinarians.

**Methodology:**

A cross-sectional study was conducted among 123 dog-owning households in thirteen fokontanys in Mahajanga from July 4 to September 13, 2016. Single and multi-member dog-owning households in the study area on the day of the interview were eligible for inclusion and purposively selected with the support of a local guide. The survey included a household questionnaire capturing information on the dog’s demographics, husbandry practices, knowledge and practices towards rabies and its control measures; the dog ecology questionnaire collected dog characteristics, vaccination status and husbandry practices. All households that reported a dog bite incident, were invited to participate in a dog bite questionnaire. In addition, direct observations of roaming dogs were conducted to assess dog population demographics and to document behavioural characteristics. Two veterinarians were purposively selected and took part in an interview during the survey period, providing information on rabies control activities, including dog-care practices in the area. Descriptive and inferential data analyses were performed using Epi Info version 7.1.5.0 (CDC Atlanta, USA).

**Results:**

We recorded a total of 400 dogs, of which 338 (84.5%) were owned amongst 123 households. More than half (67.8%) of owned dogs were between 1 to 5 years old and 95.6% were kept for guarding purposes. 45% of the surveyed dogs had free access to roam outside the premises. The majority (85.4%) of dog owners were knowledgeable that a dog bite could potentially transmit RABV to humans. 19 dog bites were reported and of these 73.6% were caused by the owner’s or a neighbour’s dog. In 6 of the 19 cases, children between 7 and 15 years of age were the victims. Dog vaccination coverage against rabies was 34% among owned dogs. Of the participants aware of a veterinarian, the majority (55/82) indicated that they accessed veterinarian services at irregular intervals. The main obstacles to vaccinations cited by dog owners were limited financial resources and difficulty accessing veterinary care.

**Conclusion:**

This study contributes to enhanced understanding of the dog ecology including canine vaccine coverage as well as knowledge and practices of dog owners in Madagascar. Most dogs in the study area were accessible for preventive vaccination through their owners, however only one third of the investigated canine population was vaccinated against rabies. Concerted national efforts towards rabies prevention and control should aim to address financial challenges and access to veterinary services.

## Introduction

Rabies is present on all continents except Antarctica, and it is considered endemic in every country in Africa [1, 2]. Rabies virus (RABV*;* species *Lyssavirus rabies)* is causing one of the oldest zoonotic diseases known to mankind, leading to fatal encephalomyelitis in animals and humans. Despite safe and effective vaccines available for use in humans and animals, dog-mediated rabies continues to represent a serious global health threat causing 99 percent of human rabies deaths [2, 3]. About 80% of people at risk reside in poor rural areas of Africa and Asia. An estimated 96% of all reported deaths occur in the population of these two continents [4]. In Africa alone, an estimated 21,476 human deaths from dog-mediated rabies (36.4% of global human rabies deaths) occur each year, resulting in a loss of 1.34 million disability-adjusted life years (DALYs) [3, 5].

Although RABV has been circulating since at least the 19^th^ century in Madagascar, there is still lack of a national rabies plan and a country-wide prevention measures [6]. Madagascar is one of the poorest among low and middle-income countries (LMICs) with more than 80% of the population below the poverty line, i.e. earning less than 1.25USD per day [7]. The socio-economic levels are very low and financial capacities particularly limited in rural areas [8]. Financial challenges and geographically distant healthcare facilities often prevent individuals seeking healthcare and the fragile health system does not have the capacity to closely monitor the spread of the disease [9, 10]. In tandem with the lack of public education and a poorly functioning program for routine diagnosis of suspected cases (animal or human), this results in a low rate of laboratory confirmation of animal and human cases and, thus, substantially underreported human disease burden [11].

Domestic dogs are Madagascar’s most prevalent RABV reservoir, therefore it makes sense to target dog-mediated rabies as the most promising and cost-effective way to lessen disease burden and preventing human rabies deaths [2]. This relies on a stable, canine population that is healthy and immunized, as well as case management and monitoring of dog bites linked to public awareness and animal disease surveillance [2]. However, with no sustainable national rabies disease prevention and control strategy currently implemented, the Malagasy government heavily relies on indiscriminate dog culling and strychnine poisoning [12].

The purpose of this study was to understand the dog ecology including rabies vaccination coverage and to conduct a survey in dog owning households assessing knowledge and practices of dog owners in the Mahajanga Urban District. The evidence generated will hopefully contribute to shedding light on current challenges to disease prevention and to informing future disease intervention strategies in Madagascar and other affected LMICs.

## Methods

### Study area and time period

A cross sectional study was conducted in Mahajanga, Madagascar. Data collection took place from July 4 to September 13, 2016.

Mahajanga is the Boeny region’s capital, one of the country’s most populated cities with around 245,000 inhabitants. It covers 57 km^2^ and is divided into 26 “fokontany” (smallest administrative unit), each with its own chief [13]. Depending on the size of the fokontany, they can be further divided into hamlets, villages, sectors or quarters [13]. The exact number of such divisions was not recorded in the 26 fokotany selected for this study.

### Sample size

A Cochran sample size formula was used to determine the minimum number of dogs to be surveyed in order to establish estimation of vaccination coverage [14]. Since the dog population in the surveyed area was unknown, an estimated 89% dog ownership was used based on a study conducted in a similar urban setting in Antananarivo, Madagascar [15]. Other estimates included a 95% confidence interval (CI) and a margin of error of 5%. A ‘single dog ownership’ or ‘multiple-dog ownership’ was assumed, and all dogs identified in each of the households were eligible for the survey. Using these parameters, a sample size of 151 households was desired. Due to time constraints for the conduct of the study, 123 households were consented and surveyed.

### Sampling scheme

A two-stage systematic random sampling method was used to select participating fokontanys and sectors. Stage one; all 26 fokontany in the district formed the sampling framework and using simple random selection method (lottery), 13 fokontany were sampled. Stage two; a similar randomization system was applied in the selection of sectors within all chosen fokontanys. A total of 13 sectors, one sector in each selected fokontany, was determined.

Chiefs of each fokontany were consulted to discuss the research and to obtain their approval granting permission to access the communities and to invite households to take part in the survey prior to the study conduct. A local guide was employed for public advocacy in each fokontany and witnessed the verbal consenting process at each household surveyed.

123 households with dogs in selected areas were purposively chosen, approached for interviews and invited to participate in a survey using three separate pre-designed questionnaires (see S1 Appendix). Household members who were available and above the age of 18 at the time of the visit were approached and one household member asked to participate in the survey on behalf of the whole household.

### Data collection and analysis

Prior to data collection, questionnaires were translated from French into local language (Malagasy) and were reviewed and discussed among investigators. The *household questionnaire* captured demographic characteristics of the dogs, husbandry practices, knowledge, and practices of household members towards rabies and its control measures. The *dog ecology questionnaire* included all dogs as reported by the household head or informant, collected characteristics of each dog (age, gender, breed), vaccination status and dog specific husbandry practices (provision of shelter, access to roaming freely). All households that reported an incident of a dog bite, were invited to take part in a *dog bite questionnaire* to capture details of the indicated bite incident.

Interviews with key informants, i.e., two resident veterinarian practitioners (the Head of the Regional Veterinary Service and a commune veterinarian officer) were also conducted to understand rabies control activities including dog-care practices in the area. In addition, direct observations of roaming dogs were conducted in the early mornings or late afternoons in the surveyed areas to assess the demographics of this dog population and to document their social behaviour. Residents and a local guide familiar with individual dogs assisted with the completion of the survey.

All collected data were verified and entered into a Microsoft excel database and were then exported into Epi Info™ version 7.1.5.0 (CDC Atlanta, USA) for detailed descriptive analysis. The Chi-square and Fischer’s exact test were used to test the relationship of selected categorical variables at 95% confidence interval.

### Ethics approval and consent to participate

Ethical approval was provided by the Faculty of Medicine at the University of Antananarivo.

## Results

### Dog ecology

We recorded a total of 400 dogs across the thirteen fokontanys surveyed. Of these, 84.5% (338/400) dogs were linked with owners within the 123 households, while the remaining were free-roaming dogs, i.e., either stray or had unknown owners (62/400). The local breed of the sub-Saharan origin was the most prevalent (84.6%, 286/338) dog breed among households surveyed. Dog ownership ranged from one to nine dogs with 52% (66/123) of households keeping more than two dogs (Table 1). The purpose of dogs was primarily reported to be guard dogs, and this was reflected in the preference for male over female dogs (ratio of 2:1).

**Table 1:** Characteristics of dog ecology.

			N (%)
Households owning dogs			123
Number of household members			759
Number of dogs per household			
	1		26 (21.1)
	2		33 (26.8)
	> 2		64 (52)
Owned dog to person ratio	1: 2		
Owned male to owned female dog	2: 1		
Household barriers	No fence nor wall		13 (10.6)
	Fence or wall but dogs can exit		50 (40.7)
	Fence or wall constituting an obstacle		60 (48.8)
Awareness of a veterinarian	Yes		82 (66.7)
	No		41 (33.3)
Distance to the nearest veterinarian (N = 82)	< 1 km		19 (23.2)
	1–2 km		37 (45.1)
	>2 km		26 (31.7)
Access to the veterinarian		Irregular	Regular
	< 1 km	10 (52.6)	9 (47.4)
	1–2 km	26 (70.3)	11 (29.7)
	>2 km	19 (73.1)	7 (26.9)

Observation of the free-roaming dogs showed that the majority were adult males (28/62), appeared lean (25/62) and were usually observed moving or lying in the company of other roaming dogs (Table 1). Notably, 18 of these free-roaming dogs exhibited aggressive behaviour (barking, growling, lunging, or biting) towards other dogs or people when approached.

In dog owning households, only 147 (43.4%) of the dogs had a collar meant for use with a leash and males were more likely to wear a collar than their female counterparts (Table 2). The majority (243/338) of the dogs were kept without kennel or other construction for shelter. Sixty households (48.8%) had some kind of enclosure to keep their dogs on the compound, while the other households allowed the dogs to freely roam. Respondents were further asked if they observed unidentifiable dogs within their compounds and such sightings were reported by 55 households (44.7%).

**Table 2:** Dog husbandry practices in Mahajanga, Madagascar.

		N (%)
Total number of owned dogs	338	
Gender and dog age (N)		
Males	< 1 year	53 (23.8)
	1–5 years	153 (68.6)
	>5 years	17 (7.6)
	Mean age (months)	31.5
Female	<1 year	25 (21.7)
	1–5 years	76 (66.1)
	>5 years	14 (12.2)
	Mean age (months)	36.5
Reason for keeping the dog (single selection)	Guarding	323 (95.6)
	Hunting	2 (.6)
	Companionship	13 (3.8)
Collar provided	Yes	147 (43.5)
	No	191(56.5)
Dog restraining practice	Always restrained	35 (10.4)
	Semi-restrained (restrained during the day)	148 (43.8)
	Free roaming	155 (45.9)
		
Shelter	In the house with the owner	18 (5.3)
	Garage	31 (9.17)
	Dedicated dog house	46 (13.6)
	No shelter at all	243 (71.9)
Vaccination status of owned dogs	Yes	115 (34.)
	No	223 (66)
Vaccination status by sex of the dog (N = 115)	Male	78 (35)
	Female	37 (32.2)
Vaccination status by age of the dog (N = 115)	Young dogs (<12 months)	8 (10.3)
	Adult dogs (>12 months)	107 (41.2)
Reasons for non-vaccination of individual dogs (n = 223)	Lack of financial means	75 (33.6)
	Lack of veterinary service	54 (24.2)
	Accessibility of veterinary service	47 (21.1)
	Lack of knowledge (owner)	34 (15. 3)
	Other	13 (5.8)
Incident of owned dog biting someone and victim of the incident	Yes	47 (13.9)
	No	291 (86.1)
	
	Owner or member of household	5 (10.6)
	Another person	42 (89.4)
Incident of owned dog been bitten by other dogs	Yes	49 (14.5)
	No	247 (73.1)
	Don’t know	42 (12.4)
Number of free-roaming dogs (N)	62	
Males	< 1 year	11 (28.2)
	>1 year	28 (71.8)
Female	< 1 year	5 (21.7)
	> 1 year	18 (78.3)
Observed body condition	Fat	37 (59.6)
	Skinny (lean)	25 (40.3)
Observed living style	Solitary	25 (40.3)
	With other dogs	37 (59.7)
Social behavior	Aggressive	18 (29)
	Docile	44 (71)
Known in the neighborhood	Yes	49 (79)
	No	13 (21)
Antiparasitic treatment	No	220 (65.1)
	Yes	118 (34.9)
	Irregularly	36 (10.7)
	Annually	12 (3.6)
	Every 6 months or more often	70 (20.7)

### Dog vaccination and care practices

**Fig 1:**
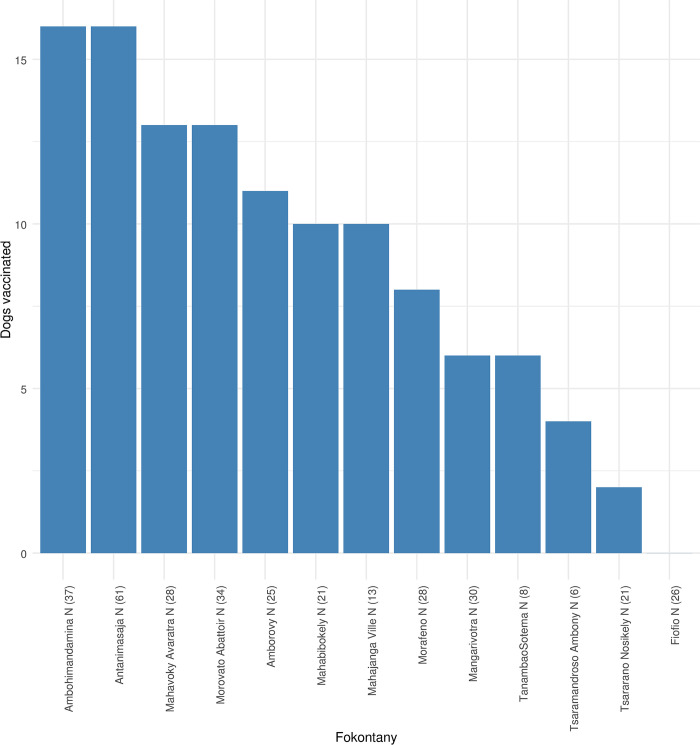
Dog vaccination status in 338 identified owned dogs across 13 districts (Fokontany) in Mahajanga commune.

In accordance with the local practice and following the manufacturer’s product information for the vaccines available in country, i.e., Rabisin^®^ (Boehringer Ingelheim, Germany) and NOBIVAC^®^ (MSD Animal Health, Boxmeer, The Netherlands), rabies immunization was considered valid up to 3 years after vaccine administration. In the surveyed households, 115 of the dogs were reportedly vaccinated (evidenced by showing the vaccine book or self-reported by the owner), resulting in vaccination coverage was at 34%. Vaccination status of dogs varied across the 13 fokontanys (Fig 1) with none of the dogs in fokontany Fiofio vaccinated. The percentage of vaccinated male dogs was higher than female dogs, but this difference was not statistically significant (*p*>.05). Statistically significantly more adult dogs (125/260) were vaccinated compared to dogs that were less than one year old (*p* < .05). The main reasons cited by owners for not vaccinating dogs were financial constraints and lack of geographical access to veterinary care (Table 2). Over 65% of dog owners were aware of the existence of a veterinarian service within their community. Of these, the majority mentioned that the nearest veterinarian was located within 1–2 km of their households. Of the participants aware of a veterinarian, the majority (55/82) indicated that they accessed veterinarian services at irregular intervals (ranging from never to when absolutely required) and the rest indicated they accessed veterinary services regularly (quarterly to once a year). When grouped by distance, 73.1% of households with irregular veterinarian access were >2km while 47.4% of households with regular veterinarian access were <1km from the next veterinarian facility. Furthermore, the reported percentage of antiparasitic treatment, considered as a surrogate for general attitude towards healthcare for dogs showed that only 82 dogs (24.3%) were treated with an antiparasitic once per year or more (2). Most dogs (57.7%, 195/338) had never been examined by a veterinarian.

### Incidence of dog bites

Dog bites were reported in 17 households with dogs (13.7%, Table 2). Over 47 owned dogs had reportedly bitten a person, 42 of these incidents involved people from outside the household Half of the dogs (24/47) that had a history of biting someone was reportedly unvaccinated. Up to 49 owned dogs (14.5%, 49/338) were purportedly attacked on one or more occasions by other known or unknown dogs whose rabies vaccination status was uncertain. Of these 65.3% (32/49) were unvaccinated.

Altogether, 19 individual dog bites experienced amongst the household survey respondents were described across the 13 fokontanys. Two of the 19 dogs that bit someone were recollected to have been killed, two had disappeared without trace, and the others remained alive. Thirteen victims of these dog bites were male, and six cases involved a child aged 7 to 15 years. Nine individuals were bitten by a neighbour’s dog, five individuals were bitten by the family dog and the remaining were either bitten by a free-roaming or unidentifiable dog.

### Rabies prevention and control measures

The household survey collected data on the knowledge about rabies, and general knowledge of rabies control and practices. Most dog owners (85.4%, 105/123) were aware of the risk to humans and dogs of contracting rabies from a bite irrespective of an incident of a dog bite incident within their household in the past. 14.6% of dog owners were either unaware of rabies or they associated dog bites as a risk for transmission of other diseases, for example plague and malaria. In addition, more than half (56.9%, 70/123) reported they would seek medical attention in the potential event of a bite in any family member, while 28% would only undertake cleaning a bite wound with soap and water. Of note, seeking immediate care for their dog from a veterinarian was only reported amongst eleven owners.

### Interviews with local veterinarians on current practices for local rabies control measures

The two (100%) interviewed veterinarians indicated that dog owners were very hesitant to request rabies vaccination and medical examination for an animal involved in a biting incident. Between the two local veterinarians, a combined total of 121 Rabisin^®^ (Boehringer Ingelheim, Germany) and NOBIVAC^®^ (MSD Animal Health, Boxmeer, Netherlands) rabies vaccines doses had been administered in 2016; however, there was no practice of marking vaccinated animals. Culling is the official policy to respond to any suspected or confirmed dog rabies case (Decret No. 95–375, 1995) and dog vaccines are limited in availability and only at a cost to owners [16]. After an event of a dog bite community members often conduct dog kills by throwing stones or lances. Annual culling events of free roaming dogs using strychnine was reported to be carried out in all 13 fokontanys, in addition it was highlighted that the veterinarians and their staff undertook annual vaccination campaigns to the communities they were serving.

## Discussion

This cross-sectional survey presents demographic data on the canine population including dog ecology and vaccination status offering an initial understanding of dog owners’ knowledge and practices related to rabies in urban Mahajanga, Madagascar.

A survey of 400 dogs in thirteen communities in Madagascar found that 84.5% of the dogs were associated with owners, with the remainder roaming free. The majority of dogs in the households were of a local breed with a sub-Saharan origin, and their primary purpose was reported to be as guard dogs, while the Coton de Tulear, which is said to have originated in Madagascar was less common in the urban district of Mahajanga [17]. Free-roaming dogs were mostly adult males, lean, and some exhibited aggressive behaviour. Few dogs wore collars meant for use with a leash, and the majority were kept without shelter or in enclosures on the compound. Over 40% of respondents reported seeing unidentifiable dogs on their property. Madagascar currently upholds the tradition of indiscriminate dog culling [16]. Dog culling and poisoning may temporarily reduce the number of dogs in a given area, but it does not address the underlying issue of unvaccinated dogs and does not prevent new dogs from entering the area and is therefore considered ineffective in achieving significant reduction of rabies infections in canine populations [18, 19]. Considering that the majority of dogs are not confined to the compound but allowed to roam freely, culling may even worsen the situation as it can lead to dog migration to avoid culls and additionally create a “vacuum effect” where new dogs move into an area to take the place of those that have been culled, potentially increasing the spread of infected dogs [20]. Further replacement of terminated dogs with unvaccinated puppies might ultimately undermine reaching high coverage [21]. As standardized laboratory, screening of samples from suspected rabid dogs are rarely conducted after their termination, it is currently difficult to estimate the actual rabies infection rate in Madagascan dog populations.

Compared with other cities in Madagascar where vaccination rates ranged from 3.3% to 17.5% [15, 22], the urban district of Mahajanga had a promising rabies vaccine coverage rate of 34% in owned dogs of households that took part in our survey. This indicates that the veterinary department’s previous public outreach efforts have helped raise awareness of the dangers of rabies infections. However, assuming that free-roaming dogs are not vaccinated, Mahajanga falls short of the 70% coverage which is considered the minimum threshold for achieving herd immunity against rabies in dogs and hence required to minimise the spread of the disease [23]. Rabies vaccination and prevention programs in vary depending on the country’s resources and level of development. However, there have been significant efforts by governments, international organizations, and non-governmental organizations (NGOs) to improve rabies control globally and a success story in particular has been Brazil [24, 25]. Vaccination programs targeting dogs are the primary method of rabies prevention in most LMICs. Conducted periodically or on a continuous basis, depending on the resources available, they have proven to be effective tools for rabies prevention and control in regions of Indonesia, South Africa and Tanzania [18, 20, 26].

Improvement in vaccine coverage in the owned dog population has recently been highlighted as critical to effective and sustainable disease control, as efforts focusing solely on stray dogs are unable to eradicate rabies in mixed populations [27]. In our survey, interviewed dog owners knew that dogs were a potential RABV vector, still only 34% of all owned dogs were vaccinated. The main barrier to vaccination appears to be the cost of vaccines. 75 dog owners reported not to have the financial resources to pay for vaccination, especially as it is not subsidized by the government or provided free of charge. In addition, availability of veterinary services has been indicated as limited by either geographical access or complete lack thereof. This can make it difficult for dog owners to access vaccines and veterinary care for their dogs. Our results are consistent with recent reports that identified key barriers to vaccination as a lack of financial resources and geographic inaccessibility to veterinary services [28, 29]. Potential to raise dog vaccination coverage in resource limited settings has been indicated by a systematic review on dog rabies vaccination coverage in Africa showing that coverage following a free-of-charge vaccination scheme was closer to the recommended 70% coverage rate (68%) than that achieved by owner-charged vaccination schemes (18%) [30].

Incidences of dog bites (among humans and dogs) were common in the urban district of Mahajanga; the most frequently reported incidents amongst survey respondents involved either a neighbour’s or the owner’s dog. Approximately one third of all reported dog bites affected children aged 7 to 15 years. This is in line with similar findings in other countries that showed 30–50% of those receiving post-exposure treatment were below 16 years of age, suggesting a particularly higher risk of contact with rabid dogs among children as they are more likely to underestimate the danger of confrontation with dogs and tend to riskier behaviour [19]. In addition, children are at higher risk of bites on their heads and faces and may fail to report incidents due to fear of punishment or lack of knowledge [19]. Owned dogs were at risk to bites by other dogs (either stray or roaming owned dogs) as about half of households were not restricting their dogs from leaving the compound. Particularly unobserved bites or contacts with potential rabid dogs that leave no marks and are hence undetected by the owners pose a risk for further transmission to the human population.

Post-exposure prophylaxis (PEP) including human rabies vaccination is a critical intervention for preventing rabies in people who have been bitten or scratched by an animal that may have rabies.

In our survey, just over one fourth of respondents (28%) agreed that treating the wound with soap and water or a disinfectant was a first-line therapy. With more than half (70/123) of the households reporting that members would intend to seek healthcare, hence receiving PEP at a healthcare facility if available, a positive trend of disease education in the population was observed. This shows that many people are aware of the threat, but further education is needed so that vulnerable individuals understand the potential consequences of failing to receive PEP which can lead to delays or failure to seek treatment [31]. This may improve immediate wound management at the individual level, however the WHO has just recently urged for Health Ministries to improve healthcare service delivery and enabling underserved population to access rabies vaccines [2]. As medical and healthcare resources are generally limited and the weak health system is a major challenge in Madagascar, improvement of existing infrastructures and expansion of the healthcare system including integrated health services, particularly in remote settings, can be a significant gamechanger for disease prevention in the future.

There were several limitations to this study. We used non-probabilistic sampling. The surveyed individuals included either dog owners or veterinarian practitioners. It is therefore likely that results on disease knowledge presented here do not fully reflect the knowledge of the general population in the study area. Generalizations of the results should be made with care since dissimilar socio-economic conditions, education levels and experiences among surveyed individuals vary across Madagascar. A recent national statistics report indicated ranking of well-being was distributed unequally in the Boeny region with a small majority for the richest groups [32]. Extensive information about the background of the research and the study team was given to reduce the bias. Furthermore, we did not reach the desired sample size, and this might affect our analyses and true reflection of the study area. In addition, we recognize challenges of estimating free-roaming dogs and methods of estimating this canine population (distance sampling and capture-recapture), however smaller study areas provided an opportunity for direct observation [33, 34]. Therefore, inaccuracies in the detection of stray dogs cannot be excluded, thus, careful consideration is recommended in interpreting the estimation of dog population.

## Conclusion and recommendations

This study enhanced understanding of the barriers to rabies transmission in both human and animal populations in Madagascar.

The study suggests that encouraging responsible dog ownership and vaccination among dog owners can significantly raise vaccination coverage to 70% or more. The main obstacle reported was limited financial means among dog owners, highlighting the need for access to affordable vaccines. Partnerships between government agencies, NGOs, and local communities can be an effective way to reach vulnerable populations and improve vaccination coverage [35]. Access to the canine population for vaccination could also be leveraged from integrating rabies vaccination into annual livestock vaccination programs currently mandated in the country and supported by the Food and Agriculture Organization by the United Nations (FAO) in response to the current food crisis in South Madagascar [36].

The surveyed dog owners lacked knowledge about rabies risk, the importance of PEP, and its proper procedures. To address this, children and dog owners would benefit from educational programs that emphasize on simple preventive measures like wound washing with water and soap, which can be done at home, even in areas with limited financial resources and healthcare access. Collaborative efforts involving the Ministry of health, the department of veterinary services in the Ministry of Agriculture, Livestock, and Fisheries, and Institute Pasteur of Madagascar (IPM) can help disseminate knowledge about rabies and its transmission [37].

## Supporting information

S1 Appendix Survey form. (DOCX)
