# Supplementary material for: Dog ecology and rabies control including canine vaccination coverage: Impacts from a survey in Madagascar
Source: PLoS One. 2024 May 9;19(5):e0302690. doi: 10.1371/journal.pone.0302690 (PMC11081359; doi:10.1371/journal.pone.0302690)
Supplement: S1 Appendix — (DOCX) [file pone.0302690.s001.docx]

**SERVY FORM - HOUSEHOLDS (General Population)**

o Fokontany: ………. Sector: …

o House Number:

o Contact:

_______________________________________________________________________________________

a) No. of people living in the household: /__/__/

b) Fence around the house:

/__/ no fence nor wall

/__/fence or wall but dogs can exit

/__/fence or wall constituting an obstacle for dogs

Distance between house and next-door neighbour:

/__/__/__/ m

c) No. of dogs in the household: /__/__/

d) No. of dogs present during our visit: /__/__/

e) Are dogs fed in your home?

/__/yes

/__/no

f) Your own dogs?

/__/yes

/__/no

/__/not concerned

g) Other dogs?

/__/yes

/__/no

h) Do dogs feed on your garbage?

/__/yes

/__/no

i) Do you sometimes find dogs strolling around your property?

/__/yes

/__/no

j) Have members of your family been bitten?

/__/yes

/__/no

If yes: FILL OUT SURVEY FORM BITES

k) What would you do in case of a dog bite?

/__/nothing

/__/clean the wound

/__/go to the doctors

/__/go to the veterinarian

/__/go to Pasteur Institute

/__/don’t know

l) Can a bite from an animal transmit diseases?

/__/yes

/__/no

/__/don’t know

m) If yes, which one do you think of first:

……………………………….

n) Have you recently seen any dead dogs around your property?

/__/yes

/__/no

o) If yes, what do you think it died of?

/__/poisoning

/__/long sickness

/__/road accident

/__/other: …

p) Do you know where the next veterinarian is located?

/__/yes

/__/no

q) If yes how far is it from your home?

/__/very far (>2km)

/__/far (1 à 2km)

/__/near (<1km)

/__/don’t know

**ANNEXE III**

**SERVY FORM – DOGS WITH OWNER**

o Fokontany: ………... Sector: …

o House Number: No. of dog

o Contact: Name of dog:

**__________________________________________________________________________________________**

General Information about dog

Sex: M / F

Age:

Race:

a) Provenance:

/__/bought

/__/it was a gift

/__/born on the property

/__/found and adopted

b) Motif for having a dog:

/__/watch dog

/__/hunt

/__/companion

/__/other: …

c) Does the dog have a collar?

/__/Yes

/__/Non

d) Is the dog marked?

/__/Yes

/__/No

e) How is the dog being kept?

/__/attached:

/__/during the day

/__/during the night

/__/ free:

/__/in the courtyard (does not leave)

/__/total freedom (leaves property)

f) Type of shelter:

/__/house

/__/garage

/__/nothing specific

/__/nothing

/__/other: ….

g) Feeding:

/__/special dog food

/__/left overs

/__/prepared by neighbours

/__/dog finds food by itself

h) Character:

/__/aggressive to all

/__/aggressive to strangers

/__/docile

i) Veterinary care:

/__/regularly (at least once a year); Frequency?

/__/irregularly

j) Has the dog ever bit someone:

/__/yes

/__/No

k) If yes who?

/__/owner or member of household

/__/_/another person. Where?

/__/in the house

/__/outside (hostile terrain)

l) did a veterinarian examine the dog?

/__/yes

/__/no

m) Has/Have your dog(s) ever been bitten by other dogs?

/__/yes – which ones

/__/no

/__/don’t know

n) If yes:

/__/by dogs on the property

/__/by the dog(s) of the neighbour

/__/by unknown stray dogs

o) Has/Have your dog(s) ever been bitten by another animal?

/__/yes; which ones?

:………………………………..

/__/no

/__/don’t know

p) In case of a dog bite, do you go to a veterinarian?

/__/yes

/__/no

q) Is/ Are your dog(s) vaccinated against rabies?

/__/yes

/__/no

r) If yes:

/__/regularly;

• date of last vaccination:

(as in vaccination record) :/__/__/__/__/__/__/

• type of vaccine: ….

/__/irregularly

s) If no vaccination or irregular vaccination – Why?

/__/ignorance

/__/not accessible

/__/financial issues

/__/no veterinarian

/__/other: …

t) cost of vaccine (visit to vet + vaccine):

/__/__/__/__/__/__/ Ariary

u) deworming:

/__/yes:

/__/systematically frequency?

/__/not systematically

/__/no

v) spaying

/__/yes

/__/no

w) If the dog is female:

- number of puppies in the las litter: …
- what happened to them?

…………………………………

…………………………………

………………………………

**ANNEXE IV**

**SERVY FORM – STRAY DOG**

o Fokontany: ……….. Sector: …

o No. of dog Breed:

o Sex M / F

1) Age:

/__/young (<12 months)

/__/adult

2) physical appearance:

/__/fat

/__/skinny

3) Way of life:

/__/solitary

/__/in a group

4) behaviour:

/__/aggressive

/__/docile

5) known in the neighbourhood:

/__/yes

/__/no

6) No. of people asked in the street about this specific animal: /__/__/

**ANNEXE V**

**SURVEY FORM – DOG BITE**

o Fokontany: ……….. Sector: ………

o House Number: Contact: o Date of the biting:

_________________________________________________________________________________________

1. General information

Place where biting took place:

Time the biting took place:

2. Was the dog provoked:

/__/yes

/__/no

3. Information about the dog:

/__/owned dog

/__/dog from the household

breed: ….

sex: ….

age: ……

/__/dog of the neighbour

breed: ….

sex: ….

age: ……

/__/stray dog

/__/non-identified dog

4. Sex of person bitten: M / F

5. Age of person bitten: /__/__/__/

6. location of bite: ………………………………………………

7. Post exposure care:

……………………………………..

……………………………………..

……………………………………..

8. Vaccination post exposure (ait IPM)

/__/yes

/__/no

9. What happened to the dog: ……………….

**ANNEXE VI**

**SURVEY FORM - VETERINARIAN**

o Fokontany: ……….. Sector: …

o Name of veterinarian:

o Contact:

o Name of veterinary clinic: …

______________________________________________________________________________________

1. Is the veterinary staff vaccinated against rabies?

/__/yes

/__/no

2. Do you have a health mandate?

/__/yes

/__/no

3. Activities in the fight against rabies

………………………………………..

4. Are biting dogs being monitored?

/__/yes

/__/no

How? ………………………………………………………………………………………………………

5. Have you ever taken samples for rabies control from an animal that bit?

/__/yes

/__/no

6. Have you ever taken samples for rabies control from a deceased animal?

/__/yes

/__/no

7. Attitude of dog owner most encountered?

/__/motivated

/__/negligent

8. Do people with a dog bite come for advice?

/__/yes

/__/no

9. Which rabies vaccine do you use? …………………………………………

10. Number of rabies vaccines sold per year: ……...

11. Number of tattoos (or other identification methods) performed on dogs per year: /__/__/__/__/
